# Serial Testing for SARS-CoV-2 and Virus Whole Genome Sequencing Inform Infection Risk at Two Skilled Nursing Facilities with COVID-19 Outbreaks — Minnesota, April–June 2020

**DOI:** 10.15585/mmwr.mm6937a3

**Published:** 2020-09-18

**Authors:** Joanne Taylor, Rosalind J. Carter, Nicholas Lehnertz, Lilit Kazazian, Maureen Sullivan, Xiong Wang, Jacob Garfin, Shane Diekman, Matthew Plumb, Mary Ellen Bennet, Tammy Hale,, Snigdha Vallabhaneni, Sarah Namugenyi, Deborah Carpenter, Darlene Turner-Harper, Marcus Booth, E. John Coursey, Karen Martin, Melissa McMahon, Amanda Beaudoin, Alan Lifson, Stacy Holzbauer, Sujan C. Reddy, John A. Jernigan, Ruth Lynfield, Minnesota Long-Term Care COVID-19 Response Group, Minnesota Long-Term Care COVID-19 Response Group, Brittney Bailey, Cory Cole, Kathy Como-Sabetti, Richard Danila, Emilio Dirlikov, Kris Ehresmann, Carrie Euerle, ; Ashley Fell, ; Rhylee Gilb, Bradley Goodwin, Kelly Hatfield, Nikki Hayes, Lisa Jacobson, ; Michelle Larson, ; Gina Liverseed, Leslie Lovett, J.P. Mahoehney, Erica Mumm, Nadia L. Oussayef, Sukarma SS. Tanwar, Sandra Turbes, Jacy Walters

**Affiliations:** ^1^CDC COVID-19 Response Team; ^2^Minnesota Department of Health; ^3^Epidemic Intelligence Service, CDC.; Minnesota Department of Health; Minnesota Department of Health; Minnesota Department of Health; Minnesota Department of Health; CDC COVID-19 Response Team; Minnesota Department of Health; Minnesota Department of Health; Minnesota Department of Health; Minnesota Department of Health; CDC COVID-19 Response Team; CDC COVID-19 Response Team; CDC COVID-19 Response Team; Minnesota Department of Health; Minnesota Department of Health; Minnesota Department of Health; Minnesota Department of Health; Minnesota Department of Health; Minnesota Department of Health; CDC COVID-19 Response Team; CDC COVID-19 Response Team; Genevive; Minnesota Department of Health

SARS-CoV-2, the virus that causes coronavirus disease 2019 (COVID-19), can spread rapidly in high-risk congregate settings such as skilled nursing facilities (SNFs) (*1*). In Minnesota, SNF-associated cases accounted for 3,950 (8%) of 48,711 COVID-19 cases reported through July 21, 2020; 35% of SNF-associated cases involved health care personnel (HCP*), including six deaths. Facility-wide, serial testing in SNFs has been used to identify residents with asymptomatic and presymptomatic SARS-CoV-2 infection to inform mitigation efforts, including cohorting of residents with positive test results and exclusion of infected HCP from the workplace (*2*,*3*). During April–June 2020, the Minnesota Department of Health (MDH), with CDC assistance, conducted weekly serial testing at two SNFs experiencing COVID-19 outbreaks. Among 259 tested residents, and 341 tested HCP, 64% and 33%, respectively, had positive reverse transcription–polymerase chain reaction (RT-PCR) SARS-CoV-2 test results. Continued SARS-CoV-2 transmission was potentially facilitated by lapses in infection prevention and control (IPC) practices, up to 12-day delays in receiving HCP test results (53%) at one facility, and incomplete HCP participation (71%). Genetic sequencing demonstrated that SARS-CoV-2 viral genomes from HCP and resident specimens were clustered by facility, suggesting facility-based transmission. Residents and HCP working in SNFs are at risk for infection with SARS-CoV-2. As part of comprehensive COVID-19 preparation and response, including early identification of cases, SNFs should conduct serial testing of residents and HCP, maximize HCP testing participation, ensure availability of personal protective equipment (PPE), and enhance IPC practices^†^ (*4*–*5*).

Interim guidance for HCP mask use and SNF visitor restriction was implemented statewide by March 31, 2020; however, during April, an increase in COVID-19 diagnoses and deaths among SNF residents in Minnesota occurred. In light of the release of CDC interim guidance on May 1 (*6*), and in an effort to improve IPC and implement facility-wide SARS-CoV-2 testing, two SNFs located in the Minneapolis-St. Paul metropolitan area contacted MDH after identifying multiple confirmed resident and HCP COVID-19 cases. During April 30–June 12, nasal, nasopharyngeal, or oral swabs were collected from residents and HCP and were tested to detect SARS-CoV-2 nucleic acid by RT-PCR, which was conducted at MDH Public Health Laboratory (MDH-PHL) and multiple commercial laboratories (*6*). After a first round of testing on April 30 and May 7 in facilities A and B, respectively, serial testing was conducted in residents every 7–10 days. HCP were offered testing services at the facility during serial testing of residents as well as whenever it was convenient to account for work schedules. Residents and HCP with positive test results were excluded from future serial testing. Starting in mid-March, HCP were screened daily for COVID-19–compatible symptoms, and symptomatic HCP were sent home per MDH and CDC guidance.^§^ Symptomatic residents and HCP were tested outside of scheduled serial testing. Data on symptoms, demographic characteristics, and HCP work assignment were collected from resident charts, MDH COVID-19 case interviews, and SNF administrator interviews. MDH and CDC provided frequent onsite IPC assessment to both facilities, including review of cohorting, hand hygiene practices, and use of PPE. Residents with positive SARS-CoV-2 test results were moved to a COVID-19 care unit within each facility, and HCP with positive test results were excluded from work for at least 10 days (*7*). Whole genome sequencing was conducted by MDH-PHL on available^¶^ specimens using previously described methods (*8*). Phylogenetic relationships, including distinct clustering of viral whole genome sequences, were inferred based on nucleotide differences via IQ-TREE, using general time reversible substitution models (*9*) as a part of the Nextstrain workflow (*10*). Descriptive analyses were conducted using R (version 3.6.1; The R Foundation). This activity was reviewed by CDC and was conducted consistent with applicable federal law and CDC policy.**

## Facility A

**FIGURE 1:**
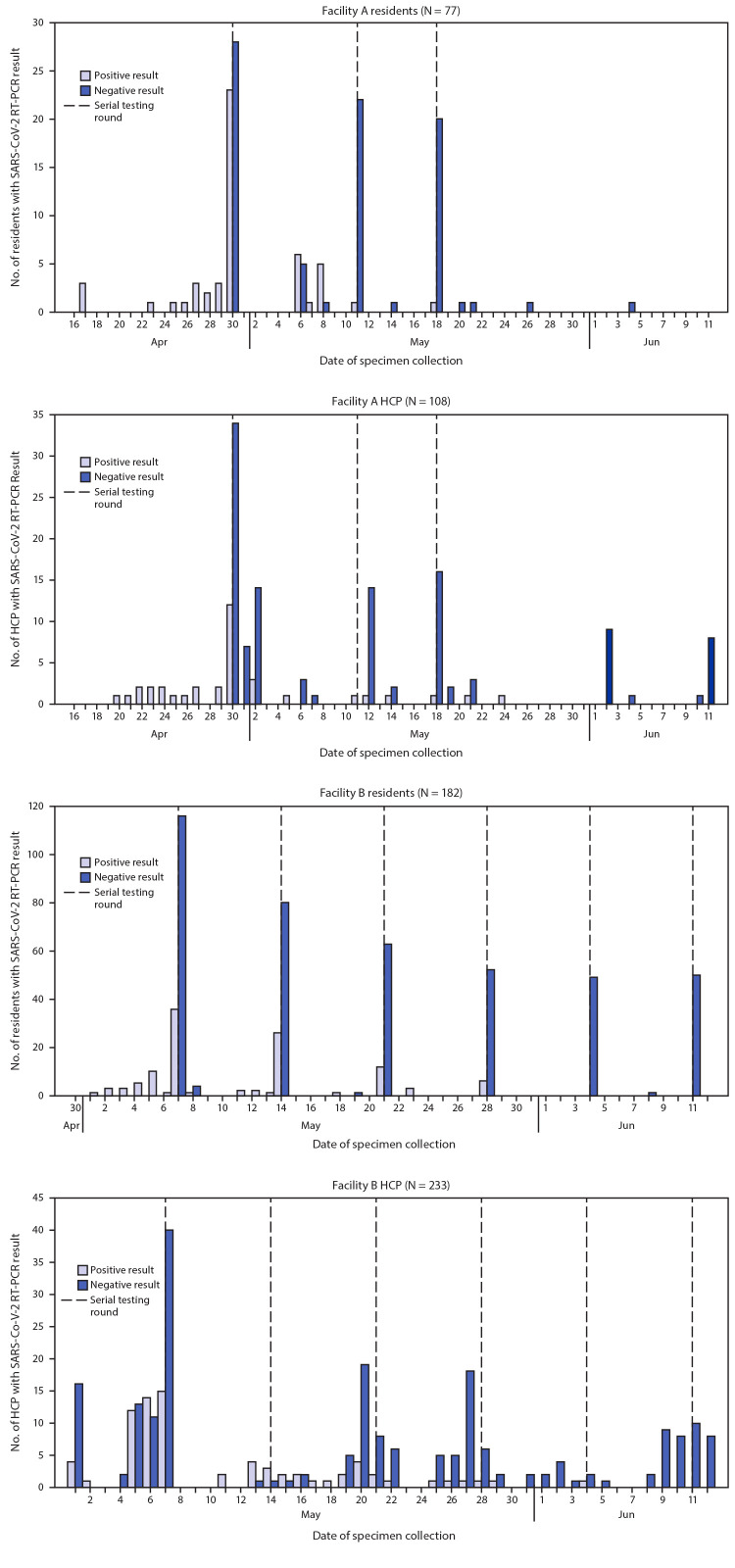
Date of serial testing round and daily specimen test results*^,†,§^ for SARS-CoV-2 detection by reverse transcription–polymerase chain reaction (RT-PCR) testing — two skilled nursing facilities, Minnesota, April–June 2020 **Abbreviation:** HCP = health care personnel. * In facility A, two residents had indeterminate results for specimens collected on April 30; one resident had a positive test result on May 7 and one resident had another indeterminate test result on May 11 before a negative test result on May 14. ^†^ In facility A, one HCP had an indeterminate test result on May 21 and was not retested. ^§^ In facility B, one resident had an indeterminate result on May 7 and had a positive test result on May 14, one resident had an indeterminate result on May 28 and had a negative test result on June 4, and one resident had an indeterminate result on June 4 and had a negative test result on June 8.

As of April 14, the census at facility A included 78 residents, with 156 HCP. Before serial testing (April 17–29), COVID-19 was laboratory-confirmed in 14 (18%) symptomatic residents. Facility A conducted three rounds of testing during April 30–May 18. During the first round of serial testing, 23 (43%) of 53 tested residents had positive SARS-CoV-2 RT-PCR test results (Figure 1); 11 refused testing. Between the first and second rounds of testing, supplementary^††^ testing of residents at risk, including nine persons who refused the first round of testing, identified 12 confirmed cases among 18 persons tested. During the second and third rounds, 4% (one of 24) and 5% (one of 21) of residents, respectively, tested positive; ongoing clinical monitoring and testing of symptomatic residents did not detect additional cases. Overall, 51 (66%) of 77^§§^ residents tested had positive test results; 14 (27%) were hospitalized and 12 (24%) died.

During April 15–29, 15 (10%) symptomatic HCP at facility A received diagnoses of confirmed COVID-19 by their health care providers (Figure 1). Among those 15 HCP, 14 (93%) worked on the third floor, where 12 of 14 residents with positive test results resided. During the first round of resident testing (April 30), specimens were collected from 43 HCP, eight (20%) of whom received a positive test result. During April 15–June 11, among 156 HCP, 108 (69%) were tested, 38 (35%) of whom had positive test results. Twenty-three (21%) HCP were tested more than once; among these, five (22%) had a positive test result after an initial negative test.

## Facility B

On April 29, the census at facility B included 183 residents with 324 HCP. Before serial testing (April 29–May 6), 24 (13%) residents had had positive SARS-CoV-2 test results after symptom onset or being tested as a roommate contact (Figure 1). Facility B conducted six rounds of testing during May 7–June 11. During the first, second, third, and fourth rounds, 24% (36 of 153), 25% (26 of 106), 16% (12 of 75), and 10% (six of 59) of residents, respectively, had positive test results. No new cases were identified among the 50 facility B residents tested in the last two rounds. Overall, among 182 residents tested, 114 (63%) COVID-19 cases were identified; 19 (17%) were hospitalized, and 40 (35%) died.

An initial round of onsite HCP testing was offered in facility B during May 1–6; 30 (42%) of 71 HCP tested on site, and one HCP tested by a primary health care provider had positive SARS-CoV-2 test results (Figure 1). Among the 31 HCP COVID-19 cases, 18 (58%) HCP worked on the first floor, where 21 (88%) of 24 infected residents were initially identified. During May 1–7, reporting of results was delayed up to 12 days for 124 HCP tested by a commercial laboratory, 44 (35%) of whom had positive SARS-CoV-2 test results; subsequently, a different laboratory was used. Overall, from May 1–June 12, 233 (72%) of 324 HCP were tested, 76 (33%) of whom had positive test results. A total of 124 (53%) results from initial HCP tests were delayed up to 12 days. Forty-nine (21%) HCP were tested more than once, including nine (18%) who had a positive test after initially testing negative.

## Characteristics of COVID-19 Cases in Health Care Personnel

Among 114 total HCP COVID-19 cases diagnosed at facilities A and B, 73 (64%) were in nurses or nursing assistants who provided direct resident care. Additional infections were identified among HCP not involved in direct care, including 13 dietary, six housekeeping, and eight social services staff members (Table). Among the 114 HCP cases, four (4%) were hospitalized, and two (2%) died. Fifty-eight (51%) persons were symptomatic on the day of testing. Among 65 HCP interviewed by MDH, 30 (46%) reported working on or after the date of their symptom onset before receiving positive test results.

**TABLE:** Demographic characteristics, symptoms, and risk characteristics of health care personnel (HCP) and residents with positive SARS-CoV-2 test results — facility A and facility B, Minnesota, April–June 2020

Characteristic	No. (%)			
	Facility A		Facility B	
	Health care personnel (N = 38)	Residents (N = 51)	Health care personnel (N = 76)	Residents (N = 114)
**Sex**				
Male	8 (21)	26 (51)	22 (29)	50 (44)
Female	30 (79)	25 (49)	53 (70)	64 (56)
Unknown	0 (—)	0 (—)	1 (1)	0 (—)
**Age, yrs**				
Median (range)	52 (18–66)	72 (33–100)	45 (17–65)	81 (52–105)
Symptomatic*^,^^†^ **on date of testing**	26 (68)	20 (39)	32 (42)	75 (66)
No symptoms*^,^^†^ **on date of testing**	12 (32)	31 (61)	44 (58)	39 (34)
Symptom onset ≤14 days after testing	0 (–)	28 (55)	2 (3)	35 (31)
Asymptomatic	6 (16)	3 (6)	3 (4)	4 (4)
**Risk behaviors/practices**				
**Worked on or after date of symptom onset^†^**				
Yes	16 (42)	N/A	14 (18)	N/A
No	12 (32)	N/A	16 (21)	N/A
Unknown/Missing	10 (26)	N/A	46 (61)	N/A
**Staff member role**				
Nurse/Certified nursing assistant	20 (53)	N/A	53 (70)	N/A
Nursing administration	1 (3)	N/A	2 (3)	N/A
Dietary	5 (13)	N/A	8 (11)	N/A
Rehabilitation	0 (—)	N/A	4 (5)	N/A
Social services	2 (5)	N/A	6 (8)	N/A
Administration	2 (5)	N/A	0 (—)	N/A
Housekeeping	3 (8)	N/A	3 (4)	N/A
Maintenance	1 (3)	N/A	0 (—)	N/A
Unknown/Missing	4 (11)	N/A	0 (—)	N/A
**Area worked/resided**				
1st floor	2 (5)	12 (24)	16 (21)	51 (45)
2nd floor	1 (3)	1 (2)	15 (20)	26 (23)
3rd floor	10 (26)	22 (43)	3 (4)	16 (14)
Multiple floors	17 (45)	0 (—)	17 (22)	12 (11)
Memory care^§^	1 (3)	16 (31)	5 (7)	9 (8)
COVID-19 unit	0 (—)	0 (—)	3 (4)	0 (—)
Unknown/Missing	7 (18)	0 (—)	17 (22)	0 (—)

**Abbreviations:** COVID-19 = coronavirus disease 2019; N/A = not applicable.

* Symptoms screening data incomplete for three residents at facility A and two residents at facility B. At facility A, one resident was discharged to another facility 2 days after a positive test result (presumed asymptomatic), one resident was evaluated at a hospital for abdominal pain and had a positive SARS-CoV-2 test result the following day (presumed asymptomatic), and one resident was evaluated at a hospital for severe chest pain and decreased oxygen saturation 4 days after a positive test result (presumed symptom onset ≤14 days after testing). At facility B, one resident was evaluated at a hospital for shortness of breath 7 days after positive SARS-CoV-2 test result (presumed symptom onset ≤14 days after testing), and one resident was admitted to hospital unresponsive with low oxygen saturation on date of testing (presumed symptomatic on date of testing).

^†^ Eight HCP at facility A and 41 HCP at facility B were not interviewed by Minnesota Department of Health. All HCP were screened for symptoms and temperature upon entering the facility and excluded if they had COVID-19–compatible symptoms; therefore, HCP with unknown or missing symptoms data who tested on the day of a facility-wide screening (six HCP at facility A and 39 HCP at facility B) were presumed asymptomatic on date of testing. HCP with unknown or missing symptoms data who were tested by their primary care provider (three HCP at facility A and three HCP at facility B) were presumed symptomatic on date of testing.

^§^ Memory care unit was located on second floor or third floor.

## Whole Genome Sequencing

**FIGURE 2:**
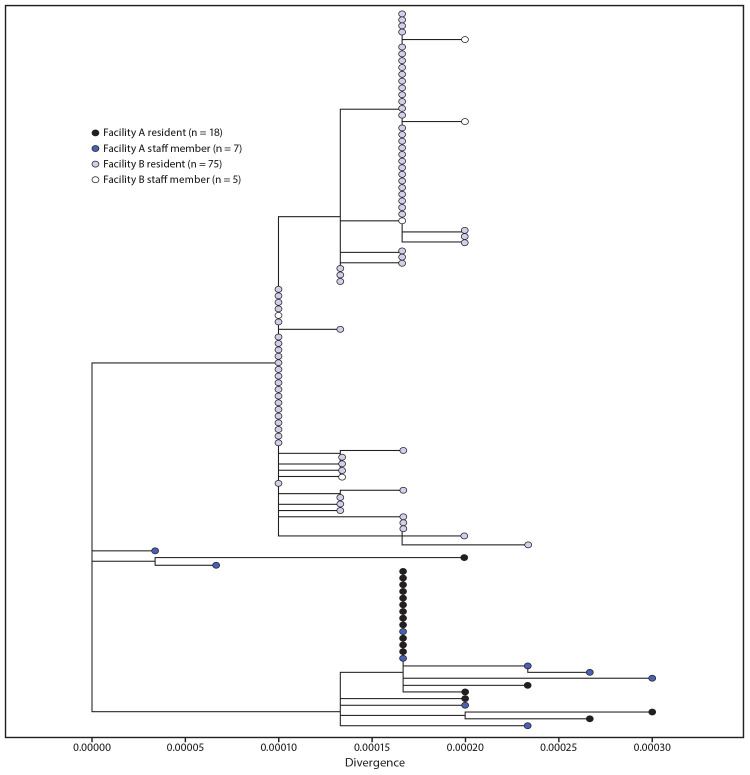
Phylogenetic trees showing genetic distance between available* SARS-CoV-2 virus specimens collected from health care personnel (HCP) and residents at facility A^†^ and facility B^§^— Minnesota, April–June 2020 * Genetic divergence based on nucleotide difference is indicated by length of branches. Available specimens included specimens tested and stored at Minnesota Public Health Laboratory and commercial labs where specimens could be retrieved and where RNA could be extracted. ^†^ Available specimens from facility A included HCP and residents diagnosed after April 29. At facility A, 17 resident and five HCP specimens had genetically similar virus strains, including one HCP with limited resident contact. Two HCP had virus sequences that were genetically different from the facility A cluster and were more similar to cases associated with community transmission in Minnesota. A third strain identified in a resident during the third testing round was genetically different from both HCP and resident strains. ^§^ Available specimens from facility B included HCP diagnosed after May 6 and residents diagnosed after April 29, throughout the outbreak. At facility B, 75 resident specimens and five HCP specimens shared genetically related strains.

Specimens from 18 (35%) residents and seven (18%) HCP at facility A were sequenced (Figure 2). Strains from 17 residents and five HCP were genetically similar, including one collected from a dietary worker with limited resident contact. Specimens from two HCP and one resident at facility A had distinctly different virus sequences from the first cluster and from each other. At facility B, 75 (66%) resident specimens and five (7%) HCP specimens were sequenced, all of which were genetically similar. The observed viral diversity of specimens associated within the two facilities was less than that observed in all sequenced specimens sampled from Minnesota cases in the community during the same period, April–June 2020 (data not shown).

## Discussion

SARS-CoV-2 transmission was decreased by early identification of asymptomatic infections through introduction of facility-wide testing and prompt implementation of mitigation efforts, including cohorting of infected residents and exclusion of infected HCP in two SNFs in Minnesota. Challenges to case identification and outbreak control included delays in reporting of test results, HCP working while symptomatic, and low baseline knowledge of and experience with IPC and PPE use. Low HCP participation in serial testing limited complete identification of infections. Anecdotal reports from HCP included anxiety about receiving positive test results, including financial losses resulting from work exclusion, and concern about workplace and community stigma.

SARS-CoV-2 viral RNA sequences isolated from HCP and residents were genetically most similar to other strains associated with the same facility, suggesting transmission within the facility. Two HCP from facility A had genetically distinct strains, highlighting the additional risk for community-acquired infections among HCP and the potential for multiple introductions. Sequence similarity among resident and HCP specimens and high rates of HCP infection, including in HCP with limited resident contact, highlight the potential for transmission between HCP or indirect routes of HCP infection from residents.

The findings in this report are subject to at least four limitations. First, symptom status might have been misclassified because case investigation data were incomplete. Second, not all eligible residents participated in each testing round, and some results were indeterminate and required follow-up repeat testing; one participant at each facility refused all testing. Third, limited participation by HCP in serial testing could have biased identification of infections and limited interpretation of genomic sequencing. Finally, whole genome sequencing was conducted on available specimens, and few specimens from the early stages of outbreaks were available, limiting the description of genetic diversity.

Serial testing of residents and all HCP, until no new cases are detected after 14 days (*4*), together with IPC strengthening, are critical strategies necessary to control COVID-19 outbreaks in SNFs. Because residents and HCP can sustain SARS-CoV-2 transmission and HCP present an ongoing risk for introducing SARS-CoV-2-from the community, barriers to HCP testing must be addressed and overcome for test-based approaches to successfully reduce COVID-19–related morbidity and mortality. HCP in SNFs are at high risk for infection, especially in outbreak settings. Testing, IPC education, flexible medical leave and PPE resources must be targeted to this at-risk workforce (*4*,*5*).

**Summary**

**What is already known about this topic?**

Facility-wide, serial testing in skilled nursing facilities (SNFs) can identify asymptomatic SARS-CoV-2 infections among health care personnel (HCP) and residents to inform mitigation efforts.

**What is added by this report?**

Serial facility-wide testing at two Minnesota SNFs identified COVID-19 cases among 64% of residents and 33% of HCP. Genetic sequencing found facility-specific clustering of viral genomes from HCP and residents’ specimens, suggesting intrafacility transmission.

**What are the implications for public health practice?**

HCP working in SNFs are at risk for infection during COVID-19 outbreaks. To protect residents and prevent SARS-CoV-2 infection among HCP, SNFs need enhanced infection prevention and control practices, assured availability of personal protective equipment, improved HCP testing participation, flexible medical leave, and timely result reporting.

## Footnotes

* HCP include, but are not limited to, emergency medical service personnel, nurses, nursing assistants, physicians, technicians, therapists, phlebotomists, pharmacists, students and trainees, contractual staff members not employed by the health care facility, and persons not directly involved in patient care, but who could be exposed to infectious agents that can be transmitted in the health care setting (e.g., clerical, dietary, environmental services, laundry, security, engineering and facilities management, administrative, billing, and volunteer personnel). HCP does not include clinical laboratory personnel.

† https://www.cms.gov/files/document/qso-20-30-nh.pdf.

§
https://www.health.state.mn.us/diseases/coronavirus/hcp/hcwrecs.pdf .

¶ Available HCP and resident specimens were those tested and stored at MDH-PHL or sent to MDH by collaborating laboratories and those from which RNA was successfully extracted.

** 45 C.F.R. part 46, 21 C.F.R. part 56; 42 U.S.C. **241(d); 5 U.S.C. **552a; 44 U.S.C. **3501 et seq.

†† Supplementary testing of nine residents who refused testing on April 30 was performed on May 5; five results were positive. Supplementary testing was performed on residents when it was discovered that two of these residents with positive test results lived in the memory care unit and had interacted with residents in that unit, and another seven cases were detected.

§§ One resident at each of facility A and B refused testing throughout the outbreak and both were treated with transmission-based precautions.
